# Spinal Drainage and Combined Pharmacotherapy as Potential Strategies to Improve Outcomes for Patients with Poor-Grade Subarachnoid Hemorrhage Treated with Clipping or Coiling but Not Receiving Nimodipine

**DOI:** 10.3390/jcm14082715

**Published:** 2025-04-15

**Authors:** Koichi Hakozaki, Fumihiro Kawakita, Kazuaki Aoki, Hidenori Suzuki, pSEED Group

**Affiliations:** Department of Neurosurgery, Mie University Graduate School of Medicine, 2-174 Edobashi, Tsu 514-8507, Mie, Japan; hako.med.xxxx@gmail.com (K.H.); fxmx0216@yahoo.co.jp (F.K.); kz.mmm.0702@gmail.com (K.A.)

**Keywords:** cerebral vasospasm, cerebrospinal fluid drainage, cilostazol, delayed cerebral ischemia, early brain injury, eicosapentaenoic acid, fasudil hydrochloride, outcome, subarachnoid hemorrhage

## Abstract

**Background/Objectives**: The outcome for aneurysmal subarachnoid hemorrhage (SAH) remains poor, particularly for patients presenting with World Federation of Neurological Surgeons (WFNS) grades IV–V. This study was designed to identify independent prognostic factors in this group of patients with poor-grade SAH. **Methods**: We prospectively analyzed 357 SAH patients with admission WFNS grades IV–V enrolled in nine primary stroke centers in Mie prefecture, Japan, from 2013 to 2022. This study compared clinical variables, including treatments for angiographic vasospasm and delayed cerebral ischemia (DCI), between patients with favorable (modified Rankin Scale [mRS] scores 0–2) and unfavorable (mRS scores 3–6) outcomes at 90 days post-onset. Multivariate analyses were then performed to identify independent determinants of favorable 90-day outcomes, followed by propensity score matching analyses. **Results**: The median age was 68 years, and 53.5% of patients had admission WFNS grade V. DCI occurred in 12.9% of patients, and 66.9% had unfavorable outcomes. Independent variables related to unfavorable outcomes were older age, admission WFNS grade V, ventricular drainage, edaravone administration, and delayed cerebral infarction, while those for favorable outcomes were spinal drainage (adjusted odds ratio [aOR] 6.118, 95% confidence interval [CI] 2.687–13.927, *p* < 0.001), modified Fisher grade 3 (aOR 2.929, 95% CI 1.668–5.143, *p* < 0.001), and triple prophylactic anti-DCI medication consisting of cilostazol, fasudil hydrochloride and eicosapentaenoic acid (aOR 1.869, 95% CI 1.065–3.279, *p* = 0.029). Nimodipine is not approved in Japan, and statin and cerebral vasospasm did not influence outcomes. As spinal drainage and the triple prophylactic anti-DCI medication were intervenable variables, propensity score matchings were performed, and they confirmed that both spinal drainage and the triple prophylactic anti-DCI medication were useful to achieve favorable outcomes. **Conclusions**: In poor-grade SAH, spinal drainage and the triple prophylactic anti-DCI medication may be effective in improving outcomes, possibly by suppressing DCI pathologies other than cerebral vasospasm.

## 1. Introduction

The outcome of aneurysmal subarachnoid hemorrhage (SAH) remains very poor, especially in patients with admission or preoperative World Federation of Neurological Surgeons (WFNS) grades IV–V [1,2], which are increasing as the population ages [3]. Delayed cerebral ischemia (DCI) is known to be an important intervening factor in determining outcomes after SAH, but its pathogenesis differs between good- and poor-grade patients [4,5]. The pathology of DCI occurring in good-grade SAH patients is almost always angiographic vasospasm (aVSP), but that in poor-grade SAH patients consists of not only aVSP but also cerebral microcirculatory disorders and neuroelectric abnormalities [6,7]. Recently, a randomized clinical trial reported that prophylactic spinal cerebrospinal fluid (CSF) drainage failed to decrease aVSP but reduced the development of delayed cerebral infarction, leading to the improvement of long-term outcomes in patients with aneurysmal SAH, including many poor-grade cases [8]. However, in actual clinical practice, perioperative management is often the same regardless of whether the clinical grade is good or poor, and therefore, the authors consider that it is meaningful to examine the treatment outcomes and outcome-determining factors of poor-grade SAH patients in specific regions.

The American Heart Association guidelines recommend only nimodipine as a prophylactic drug for DCI [9]. Nimodipine is not approved in Japan, but there are a number of medications available exclusively in Japan for DCI instead [10]. Representative agents include cilostazol (a selective phosphodiesterase type III inhibitor and antiplatelet agent), fasudil hydrochloride (a Rho-kinase inhibitor), eicosapentaenoic acid (EPA: an omega-3 polyunsaturated fatty acid), ozagrel sodium (a thromboxane A2 synthase inhibitor), and edaravone (a free radical scavenger), which are expected to have vasodilatory as well as pleiotropic properties and may be administered alone or in combination at the discretion of the neurosurgeon [10]. As DCI pathologies other than aVSP become more important in poor-grade cases [6], it is thought to be meaningful to examine the usefulness of these drugs in such cases. In April 2022, a selective endothelin receptor subtype-A antagonist, clazosentan, was clinically available in Japan, ahead of the rest of the world [11]. Clazosentan is effective against aVSP, but precautions should be taken with side effects associated with fluid retention [12]. In order to minimize clazosentan’s side effects and maximize its efficacy, each facility strives to establish optimal protocols, including indications for administration and concomitant medications [13,14]. Therefore, in this study, the authors analyzed clinical data up until the introduction of clazosentan to investigate outcomes and outcome determinants including medications in poor-grade SAH patients in Mie prefecture, a rural area of Japan.

## 2. Materials and Methods

### 2.1. Study Design

This was a retrospective study in which prospectively collected clinical data were analyzed. The study received approval from our institute’s ethics committee (approval number H2018-031), and the requirement for informed consent was waived with opt-out.

**Figure 1:**
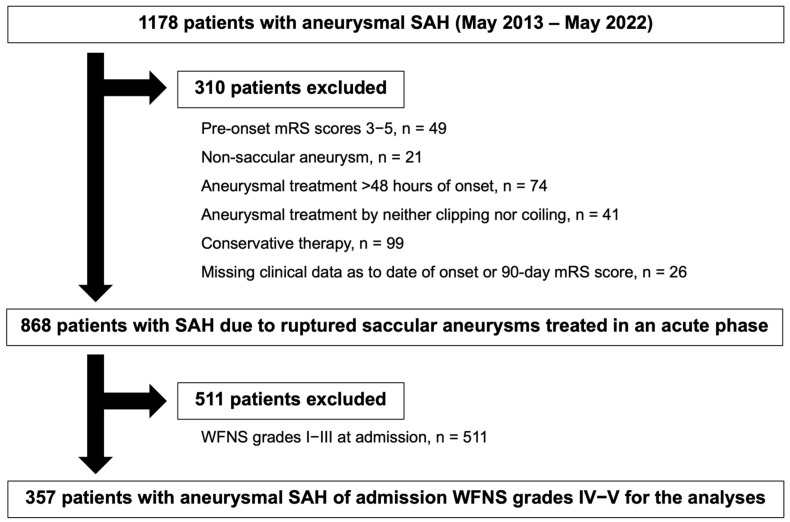
Flowchart showing the included and excluded patients in this study. SAH, subarachnoid hemorrhage; mRS, modified Rankin Scale; WFNS, World Federation of Neurological Surgeons.

The Prospective Registry for Searching Mediators of Neurovascular Events After Aneurysmal Subarachnoid Hemorrhage (pSEED) [4], which was conducted at nine primary stroke centers in Mie prefecture in Japan from May 2013 to May 2022, registered 1178 patients with SAH by ruptured intracranial aneurysms. All patients were ≥20 years of age at onset and had SAH identified on computed tomography (CT) scans or by lumbar puncture, of which the cause was diagnosed to be a ruptured aneurysm using digital subtraction angiography. In the registry, the timing of aneurysmal obliteration, selection of treatment modalities, and other medical management or treatment strategies were not limited and were determined by the treating neurosurgeons according to their preferences. In this study, 310 patients were excluded to investigate the effects of prophylactic treatments on DCI and outcomes, as shown in Figure 1. Furthermore, 511 patients with WFNS grades I–III at admission were excluded from this study. Thus, the study population consisted of 357 aneurysmal SAH patients with admission WFNS grades IV–V, whose ruptured aneurysms were treated within 48 h of SAH onset (Figure 1).

In this study, favorable and unfavorable outcomes were defined as modified Rankin Scale (mRS) scores 0–2 and 3–6, respectively, at 90 days after SAH onset. Clinical variables were compared between patients with favorable and unfavorable outcomes, followed by multivariate analyses to find independent determinants of outcomes.

### 2.2. Analyzed Clinical Variables

The baseline variables consisted of age, sex, past history of SAH by a ruptured aneurysm and cerebral infarction due to any cause, presence of pre-onset comorbidities, current smoking, location of ruptured aneurysms, WFNS and modified Fisher grades [15] at admission, and presence of acute hydrocephalus. Acute hydrocephalus was diagnosed by ventriculomegaly on admission CT scans, which was considered to be a cause of disturbance of consciousness. Treatment-related variables included treatment modalities of aneurysmal obliteration, procedural complications, CSF drainage, perioperative medications to prevent DCI, development of aVSP, endovascular treatment for symptomatic aVSP (intra-arterial fasudil hydrochloride or percutaneous transluminal angioplasty [PTA]), DCI, delayed cerebral infarction, and shunt-dependent hydrocephalus (SDHC). Procedural complications were defined as cerebral contusion, infarction, and any intracranial bleeding, which were diagnosed on CT scans or magnetic resonance (MR) images the day after surgical clipping or endovascular coiling. CSF drainage was performed to manage acute or persistent hydrocephalus and/or to promote subarachnoid blood clearance. Prophylactic anti-DCI medications included intravenous administration of fasudil hydrochloride, low molecular dextran, ozagrel sodium, and edaravone, as well as oral or enteral administration of cilostazol, EPA, statin, and mineralocorticoid. Because the combined administration of multiple drugs (e.g., cilostazol and fasudil hydrochloride) for the prevention of DCI is common in Japan, we also analyzed drug combinations that showed significant intercorrelations. Although there has been an increase in the use of antiepileptic drugs such as perampanel to treat post-SAH neuroelectric abnormalities in recent years [16], the registry did not contain information on antiepileptic drug use, and therefore the analysis of antiepileptic drug was not included in this study. aVSP was defined as more than 50% narrowing in the baseline diameter of the intracranial internal carotid artery, the main trunk of the anterior cerebral artery, middle cerebral artery, and posterior cerebral artery (A1–2, M1–2, and P1–2 segments, respectively), the vertebral artery, and/or the basilar artery, regardless of clinical symptoms. DCI was diagnosed based on a worsening neurological status due to non-iatrogenic cerebral ischemia lasting for more than one hour. All cases of aVSP and DCI were treated with induced hypertension, but the extent and specific method were determined at each institution. Delayed cerebral infarction was defined as a newly developed cerebral infarction on CT scans or MR imaging which was not detected on the day after the operation or intervention. SDHC was recorded for progressive ventriculomegaly with neurological deterioration that developed after 14 days post-SAH and was treated with CSF shunting.

### 2.3. Statistical Analysis

SPSS version 29.0 (IBM Corp., Armonk, New York, NY, USA) was used to perform statistical analyses. Variables were recorded as continuous or categorical variables. As a continuous variable, age was non-normally distributed on the Shapiro–Wilk test and was reported as medians and interquartile ranges (IQRs). Numbers (percentages) were used to report categorical variables. Univariate analyses were performed using the Mann–Whitney U test for continuous variables and using chi-square or Fisher’s exact tests for categorical variables, as appropriate. A *p* value < 0.05 was considered significant.

Prophylactic anti-DCI drugs and candidate variables for multivariate analyses were evaluated using Spearman’s correlation coefficient (ρ) for both continuous and ordinal variables or the phi coefficient (φ) for two binary variables. Correlations of |ρ| or |φ| > 0.3 were judged significant. Multivariate logistic regression analyses were performed to find independent variables for 90-day favorable outcomes by using variables with a *p* value lower than 0.05 on univariate analyses: among similar clinical variables that were significantly intercorrelated, only the variables with the smallest *p* values were used as candidate variables. Adjusted odds ratios and 95% confidence intervals were calculated.

To reduce potential selection bias, propensity score matching was performed using a multivariable logistic regression model, with exposure to independent treatment factors as binary variables and multiple factors, including age, admission WFNS grade, modified Fisher grade, treatment modality, CSF drainage, and DCI prophylaxis, selected as covariates. After the propensity score was calculated, patients were assigned to 1:1 nearest-neighbor matching without replacement using a caliper width of 0.2 standard deviations. Clinical characteristics were compared between the two groups.

## 3. Results

### 3.1. Study Population

This study analyzed 357 consecutive aneurysmal SAH patients with admission WFNS grades IV–V. The baseline characteristics of the study population are summarized in the “Overall” column of Table 1. The median age was 68 years (IQR, 56.0–77.0), and the population consisted of 264 female patients (73.9%), 191 patients (53.5%) with admission WFNS grade V, 225 patients (63.0%) with modified Fisher grade 4, and 133 patients (37.3%) with acute hydrocephalus. Ruptured aneurysms were obliterated with microsurgical clipping in 253 patients (70.9%) and simple endovascular coiling in 104 patients (29.1%), associated with treatment complications in 85 patients (23.8%; cerebral infarction in 71 patients and cerebral contusion in 14 patients). CSF drainage was performed in 167 patients (46.8%). DCI and aVSP occurred in 46 (12.9%) and 93 (26.1%) patients, respectively, and all were treated with induced hypertension. In addition, 11 patients (3.1%) were treated with intra-arterial administration of fasudil hydrochloride, and two patients (0.6%) with PTA. Consequently, 80 patients (22.4%) had delayed cerebral infarction diagnosed on CT scans (49 patients, 13.7%) or MR imaging (31 patients, 8.7%). SDHC developed in 120 patients (33.6%), and 239 patients (66.9%) had 90-day unfavorable outcomes.

### 3.2. Univariate Analyses to Detect Variables Potentially Related to Outcomes

Univariate analyses were performed to compare clinical variables between patients with 90-day favorable and unfavorable outcomes. Unfavorable outcomes were associated with significantly older age (*p* < 0.001), higher rates of concomitant dyslipidemia (*p* = 0.034), admission WFNS grade V (*p* < 0.001), modified Fisher grade 4 (*p* < 0.001), and the presence of acute hydrocephalus (*p* = 0.020), while favorable outcomes were more frequent in patients with admission WFNS grade IV (*p* < 0.001) and modified Fisher grade 3 (*p* < 0.001; Table 1). Treatment modalities for aneurysmal obliteration were not different between the two groups, but patients with unfavorable outcomes were more frequently treated with ventricular drainage (*p* = 0.012) and less frequently with spinal drainage (*p* = 0.004). Regarding prophylactic anti-DCI medications, patients with consequently unfavorable outcomes were less frequently treated with cilostazol (*p* = 0.005), fasudil hydrochloride (*p* < 0.001), and EPA (*p* = 0.017), while edaravone (*p* = 0.007) was more frequently administered to consequently unfavorable outcome patients. Unfavorable outcomes were associated with more frequent incidences of delayed cerebral infarction (*p* < 0.001) and SDHC (*p* = 0.005), although the incidence of DCI and aVSP was not significantly different between the favorable and unfavorable outcome groups (Table 1).

As to prophylactic anti-DCI medications, there were the following two significant intercorrelations: between cilostazol and fasudil hydrochloride (φ = 0.498), and between cilostazol and EPA (φ = 0.312). In contrast, edaravone showed no correlation with any of the drugs. Therefore, for the three drugs that showed a correlation, univariate analyses were performed to examine which combination had the greatest effect on outcomes. As a result, it was revealed that only triple therapy consisting of cilostazol, fasudil hydrochloride, and EPA was significantly more frequently used in the favorable outcome group (*p* = 0.015; Table 2).

### 3.3. Independent Variables Related to Favorable Outcomes

In the clinical variables investigated in this study (Table 1 and Table 2), variables with a *p* value of less than 0.05 on univariate analyses were age, dyslipidemia, admission WFNS grades IV and V, modified Fisher grades 3 and 4, acute hydrocephalus, ventricular drainage, spinal drainage, the triple prophylactic anti-DCI medication, edaravone use, delayed cerebral infarction on CT scans and/or MR imaging, and SDHC. Among these variables, there were significant correlations between WFNS grades IV and V (φ = −1), between modified Fisher grades 3 and 4 (φ = −0.906), between acute hydrocephalus and ventricular drainage (φ = 0.546), and between delayed cerebral infarction on all images and that on CT scans or MR imaging (φ = 0.742 and 0.574, respectively). Among these significantly correlated variables, those with the lowest *p* values on univariate analyses, that is, admission WFNS grade V, modified Fisher grade 3, ventricular drainage, and delayed cerebral infarction on all images, were used as candidate variables for the following multivariate analyses, in addition to the six variables that did not correlate with any other variable.

Multivariate analyses were performed to find independent variables associated with 90-day favorable outcomes using a total of ten variables selected based on univariate analyses (Table 3), or using eight variables, excluding two outcome measures from a total of ten variables selected based on univariate analyses (Table 4). Both multivariate analyses revealed that spinal drainage, modified Fisher grade 3, and the triple prophylactic anti-DCI medications consisting of cilostazol, fasudil hydrochloride, and EPA were independent variables for favorable outcomes.

### 3.4. Effects of Spinal Drainage or the Triple Prophylactic Anti-DCI Medications on Outcomes

Both spinal drainage and the triple prophylactic anti-DCI medications consisting of cilostazol, fasudil hydrochloride, and EPA were intervening variables among independent variables for 90-day favorable outcomes. First, thus, propensity score matching was performed using a multivariate logistic regression model with exposure to spinal drainage as a binary variable and age, WFNS grade at admission, modified Fisher grade, and treatment modality as covariates (Table 5). Next, we performed propensity score matching using exposure to the triple prophylactic anti-DCI medications as a binary variable and age, WFNS grade at admission, modified Fisher grade, treatment modality, CSF drainage, and other prophylactic anti-DCI drugs, including statin, low molecular dextran, ozagrel sodium, edaravone, and mineralocorticoid, as covariates (Table 6). The propensity score matching analyses showed that both spinal drainage and the triple prophylactic anti-DCI medications consisting of cilostazol, fasudil hydrochloride, and EPA were significantly associated with favorable outcomes.

## 4. Discussion

This study found that spinal drainage and the combination of three prophylactic anti-DCI medications consisting of cilostazol, fasudil hydrochloride, and EPA are independent intervenable variables for favorable outcomes in patients with admission WFNS grades IV and V SAH. Unfavorable outcomes were related to delayed cerebral infarction but not aVSP, while both spinal drainage and the triple prophylactic anti-DCI medications failed to reduce the incidence of delayed cerebral infarction according to propensity score matching. This suggests that spinal drainage and the triple prophylactic anti-DCI medications may improve outcomes by reducing the severity of delayed cerebral infarction, rather than reducing its frequency. Ventricular drainage and administration of the free radical scavenger edaravone were also associated with unfavorable outcomes: the former may reflect the effects of acute hydrocephalus due to massive bleeding requiring intervention rather than the drainage procedure itself, and the latter may have been administered to treat DCI or delayed cerebral infarction rather than to prevent them, but this could not be proven as supporting data were not registered in the database.

The pathology of DCI includes aVSP, aVSP-unrelated cerebral microcirculatory disorders, and neuroelectric abnormalities [6,7]. In patients with poorer grade SAH, DCI occurs more frequently [4], but it is thought that pathologies other than aVSP become more important as the cause of DCI [6]. Thus, depending on the patient’s pathology, some drugs were reported to inhibit aVSP without improving outcomes [17,18,19], while other drugs were reported to improve outcomes without inhibiting aVSP [20,21,22].

Blood degradation products in the subarachnoid space are believed to be an important causative agent of DCI [6], and therefore, early removal of SAH has been considered a potential preventive treatment against DCI [23]. However, the addition of lumbar spinal drainage to standard treatment reduced the incidence of DCI but did not reduce delayed cerebral infarction or improve outcomes in patients with WFNS grades I–III SAH [23]. In contrast, the EARLYDRAIN trial, a randomized clinical study in patients with SAH in which at least 40% of patients had WFNS grades IV–V, demonstrated that the addition of lumbar drainage to standard care did not improve aVSP but reduced the incidence of delayed cerebral infarction at discharge and the rate of unfavorable outcomes after six months [8]. It was speculated that lumbar drainage reduced intracranial pressure spikes, thereby suppressing the occurrence of spreading depolarizations; as a result, delayed cerebral infarction was prevented to improve clinical outcomes, highlighting the importance of intracranial pressure management in patients with WFNS grades IV–V SAH [8,24].

As to drugs, as nimodipine is not approved in Japan, alternative pharmacologic agents are used for DCI prevention. Cilostazol, a selective phosphodiesterase type III inhibitor and antiplatelet agent, possesses pleiotropic properties such as the suppression of lipid peroxidation, inflammation, endothelial damage, spreading depolarization, and tenascin-C upregulation, and has been reported to attenuate post-SAH microcirculatory dysfunction [25,26,27]. Meta-analyses of clinical trials have demonstrated that cilostazol improves DCI and patient outcomes [28,29,30,31]. Fasudil hydrochloride, a Rho-kinase inhibitor, has been widely used to prevent aVSP and DCI in Japan. It is reported to inhibit the smooth muscle constriction of cerebral artery and endothelial damage, thereby increasing the expression of endothelial nitric oxide synthase and the generation of nitric oxide, as well as to suppress leukocyte infiltration, production of inflammatory mediators and free radicals, and apoptosis [32]. EPA, an omega-3 polyunsaturated fatty acid, not only prevents aVSP via inhibition of the sphingosylphosphorylcholine-Rho-kinase pathway [33,34] but also exerts anti-inflammatory effects by inhibiting eicosanoid and cytokine production and mild antiplatelet aggregation effects [35]. A prospective, multicenter, randomized trial showed that EPA reduced symptomatic vasospasm and delayed cerebral infarction, although it did not improve functional outcomes [33].

Combining multiple drugs may provide synergistic effects and potentially enhance therapeutic efficacy, but there are limited studies reporting the potential effects of drug combinations on DCI after SAH. A Japanese nationwide survey identified that a multidrug combination consisting of cilostazol, fasudil hydrochloride, and statin was the most effective in reducing aVSP with DCI, although the survey was limited by the lack of patient-specific clinical data including SAH severity and cerebral vasospasm management, as well as the low survey collection rate of approximately 30% [10]. One study reported a beneficial effect when fasudil hydrochloride was added to a thromboxane synthase inhibitor, ozagrel sodium [36], whereas others found no additional benefit from the combination of fasudil hydrochloride and ozagrel sodium [37]. Another study found that the administration of EPA and fasudil hydrochloride reduced the frequency of aVSP and delayed cerebral infarction caused by aVSP, leading to improved clinical outcomes without an increased risk of adverse events [34]. Several studies have also reported the potential benefits of combining cilostazol and fasudil hydrochloride [4,10], which may be effective in preventing both aVSP and early brain injury-induced microcirculatory disturbances [38,39,40]. In our study, although spinal drainage and the triple prophylactic anti-DCI medications did not significantly reduce the incidence of delayed cerebral infarction in propensity score matching analyses, they were associated with a significant improvement in functional outcomes. The combination therapy may have reduced the severity of delayed cerebral infarctions by aVSP-unrelated pathologies.

This study has several limitations. First, although the data were prospectively collected across multiple centers in one region of Japan, the analyses were conducted retrospectively. Treatment strategies were determined by the attending neurosurgeons at each participating center, and treatment allocation was not controlled: the non-randomized and non-blinded interventions may have influenced the association between treatment factors and clinical outcomes. In addition, confounders not measured or assessed, such as aneurysm rerupture or recurrent SAH, might also contribute to this association. To avoid these biases, randomized controlled trials are needed. Second, the standard of care for SAH, including anti-DCI medication, differs from that in other countries. Consequently, our findings may not be directly applicable to regions with differing treatment protocols and patient demographics, and caution is needed when extrapolating these findings to other populations. Third, the limitations of this study include the lack of use of nimodipine, which is not approved in Japan, and clazosentan, a selective endothelin receptor subtype-A antagonist approved in Japan in 2022. Fourth, this study did not investigate the role of antiepileptic drugs, which may be effective in modulating the neuroelectric mechanisms of DCI [16]. Lastly, in this study, MR imaging was not performed in all patients, so the effects of spinal drainage and the triple prophylactic anti-DCI medications on the frequency and number of microinfarctions that cannot be detected by CT scans and can be detected only by MR imaging could not be evaluated. The efficacy of these treatments, including their mechanisms, needs to be further investigated.

## 5. Conclusions

This study found spinal drainage and a combination of three prophylactic anti-DCI medications—cilostazol, fasudil hydrochloride, and EPA—as independent and potentially modifiable factors associated with favorable functional outcomes in patients with poor-grade SAH. While these interventions did not significantly reduce the incidence of delayed cerebral infarction, they may have improved outcomes by attenuating the severity of delayed cerebral infarction, particularly through mechanisms unrelated to angiographic vasospasm. These findings suggest that comprehensive management strategies addressing diverse DCI pathologies may be beneficial in patients with poor-grade SAH.

## Figures and Tables

**Table 1:** Comparison of variables between patients with 90-day favorable and unfavorable outcomes.

Variables	Overall (*n* = 357)	Favorable (*n* = 118)	Unfavorable (*n* = 239)	*p* Value
Age (years), median (IQR)	68 (56, 77)	60 (49, 70)	72 (62, 80)	<0.001 ^a^
Female	264 (73.9%)	93 (78.8%)	171 (71.5%)	0.141 ^b^
Past history				
SAH	13 (3.6%)	4 (3.4%)	9 (3.8%)	1.000 ^c^
Cerebral infarction	17 (4.8%)	2 (1.7%)	15 (6.3%)	0.066 ^c^
Comorbidity				
Hypertension	162 (45.4%)	53 (44.9%)	109 (45.6%)	0.902 ^b^
Diabetes mellitus	31 (8.7%)	6 (5.1%)	25 (10.5%)	0.110 ^c^
Dyslipidemia	50 (14.0%)	10 (8.5%)	40 (16.7%)	0.034 ^b^
Current smoking	70 (19.6%)	24 (20.3%)	46 (19.2%)	0.807 ^b^
WFNS grade				
IV	166 (46.5%)	74 (62.7%)	92 (38.5%)	<0.001 ^b^
V	191 (53.5%)	44 (37.3%)	147 (61.5%)	<0.001 ^b^
Modified Fisher grade				
1	4 (1.1%)	2 (1.7%)	2 (0.8%)	0.602 ^c^
2	12 (3.4%)	3 (2.5%)	9 (3.8%)	0.757 ^c^
3	116 (32.5%)	57 (48.3%)	59 (24.7%)	<0.001 ^b^
4	225 (63.0%)	56 (47.5%)	169 (70.7%)	<0.001 ^b^
Acute hydrocephalus	133 (37.3%)	34 (28.8%)	99 (41.4%)	0.020 ^b^
Aneurysm location				
Anterior circulation	317 (88.8%)	104 (88.1%)	213 (89.1%)	0.781 ^b^
Posterior circulation	40 (11.2%)	14 (11.9%)	26 (10.9%)	0.781 ^b^
Treatment modality				
Endovascular coiling	104 (29.1%)	40 (33.9%)	64 (26.8%)	0.164 ^b^
Surgical clipping	253 (70.9%)	78 (66.1%)	175 (73.2%)	0.164 ^b^
Coiling/clipping-related complication				
Total	85 (23.8%)	24 (20.3%)	61 (25.5%)	0.279 ^b^
Cerebral contusion	14 (3.9%)	3 (2.5%)	11 (4.6%)	0.403 ^c^
Cerebral infarction	71 (19.9%)	21 (17.8%)	50 (20.9%)	0.487 ^b^
Cerebrospinal fluid drainage				
Total	167 (46.8%)	50 (42.4%)	117 (49.0%)	0.241 ^b^
Ventricular	126 (35.3%)	31 (26.3%)	95 (39.7%)	0.012 ^b^
Cisternal	43 (12.0%)	12 (10.2%)	31 (13.0%)	0.444 ^b^
Spinal	49 (13.7%)	25 (21.2%)	24 (10.0%)	0.004 ^b^
Prophylaxis for DCI				
Cilostazol	288 (80.7%)	105 (89.0%)	183 (76.6%)	0.005 ^b^
Fasudil hydrochloride	312 (87.4%)	114 (96.6%)	198 (82.8%)	<0.001 ^c^
Eicosapentaenoic acid	201 (56.3%)	77 (65.3%)	124 (51.9%)	0.017 ^b^
Statin	148 (41.5%)	51 (43.2%)	97 (40.6%)	0.635 ^b^
Low molecular dextran	159 (44.5%)	60 (50.8%)	99 (41.4%)	0.092 ^b^
Ozagrel sodium	29 (8.1%)	11 (9.3%)	18 (7.5%)	0.560 ^b^
Edaravone	99 (27.7%)	22 (18.6%)	77 (32.2%)	0.007 ^b^
Mineralocorticoid	16 (4.5%)	5 (4.2%)	11 (4.6%)	1.000 ^c^
DCI	46 (12.9%)	11 (9.3%)	35 (14.6%)	0.158 ^b^
Angiographic vasospasm	93 (26.1%)	31 (26.3%)	62 (25.9%)	0.947 ^b^
Endovascular treatment for vasospasm				
Intra-arterial fasudil hydrochloride	11 (3.1%)	5 (4.2%)	6 (2.5%)	0.516 ^c^
PTA	2 (0.6%)	1 (0.8%)	1 (0.8%)	0.552 ^c^
Delayed cerebral infarction	80 (22.4%)	10 (8.5%)	70 (29.3%)	<0.001 ^b^
On CT	49 (13.7%)	6 (5.1%)	43 (18.0%)	<0.001 ^c^
On MR imaging	31 (8.7%)	4 (3.4%)	27 (11.3%)	0.015 ^c^
SDHC	120 (33.6%)	28 (23.7%)	92 (38.5%)	0.005 ^b^

Data are numbers of patients (% of total patients per group) unless otherwise specified. *p* values: ^a^ Mann–Whitney U test, ^b^ chi-square test, or ^c^ Fisher’s exact test. CT, computed tomography; DCI, delayed cerebral ischemia; IQR, interquartile range; MR, magnetic resonance; PTA, percutaneous transluminal angioplasty; SAH, subarachnoid hemorrhage; SDHC, shunt-dependent hydrocephalus; WFNS, World Federation of Neurological Surgeons.

**Table 2:** Comparison of the three prophylactic drugs and the combinations for delayed cerebral ischemia between patients with 90-day favorable and unfavorable outcomes.

Variables	Overall (*n* = 357)	Favorable (*n* = 118)	Unfavorable (*n* = 239)	*p* Value
CIL alone	5 (1.4%)	0 (0.0%)	5 (2.1%)	0.175 ^a^
FAS alone	24 (6.7%)	6 (5.1%)	18 (7.5%)	0.502 ^a^
EPA alone	4 (1.1%)	1 (0.8%)	3 (1.3%)	1.000 ^a^
CIL + FAS without EPA	99 (27.7%)	34 (28.8%)	65 (27.2%)	0.748 ^b^
FAS + EPA without CIL	13 (3.6%)	5 (4.2%)	8 (3.3%)	0.765 ^a^
EPA + CIL without FAS	8 (2.2%)	2 (1.7%)	6 (2.5%)	1.000 ^a^
CIL + FAS + EPA	176 (49.3%)	69 (58.5%)	107 (44.8%)	0.015 ^b^

Data are numbers of patients (% of total patients per group). *p* values: ^a^ Fisher’s exact test, or ^b^ chi-square test. CIL, cilostazol; EPA, eicosapentaenoic acid; FAS, fasudil hydrochloride.

**Table 3:** Multivariate logistic regression analyses to find independent variables associated with favorable outcomes in patients with poor-grade subarachnoid hemorrhage using a total of ten variables selected based on univariate analyses.

Variables	Adjusted Odds Ratio	95% Confidence Interval	*p* Value
Spinal drainage	6.118	2.687–13.927	<0.001
Modified Fisher grade 3	2.929	1.668–5.143	<0.001
CIL + FAS + EPA	1.869	1.065–3.279	0.029
Age	0.943	0.924–0.964	<0.001
WFNS grade V	0.545	0.317–0.937	0.028
Ventricular drainage	0.527	0.284–0.977	0.042
Edaravone	0.453	0.235–0.874	0.018
Delayed cerebral infarction	0.204	0.090–0.466	<0.001
SDHC	0.617	0.321–1.186	0.148
Dyslipidemia	0.544	0.219–1.349	0.189

CIL, cilostazol; EPA, eicosapentaenoic acid; FAS, fasudil hydrochloride; SDHC, shunt-dependent hydrocephalus; WFNS, World Federation of Neurological Surgeons.

**Table 4:** Multivariate logistic regression analyses to find independent variables associated with favorable outcomes in patients with poor-grade subarachnoid hemorrhage using eight variables, excluding two outcome measures, from a total of ten variables selected based on univariate analyses.

Variables	Adjusted Odds Ratio	95% Confidence Interval	*p* Value
Spinal drainage	4.444	2.090–9.448	<0.001
Modified Fisher grade 3	3.129	1.814–5.398	<0.001
CIL + FAS + EPA	1.859	1.089–3.174	0.023
Age	0.944	0.926–0.964	<0.001
WFNS grade V	0.504	0.299–0.849	0.010
Ventricular drainage	0.482	0.270–0.861	0.014
Edaravone	0.369	0.196–0.692	0.002
Dyslipidemia	0.564	0.235–1.353	0.200

CIL, cilostazol; EPA, eicosapentaenoic acid; FAS, fasudil hydrochloride; WFNS, World Federation of Neurological Surgeons.

**Table 5:** Clinical characteristics in propensity score-matched patients with poor-grade subarachnoid hemorrhage (SAH) treated with or without spinal drainage (SD).

Variables	Overall (*n* = 98)	SD (*n* = 49)	No SD (*n* = 49)	*p* Value
Age (years), median (IQR)	69 (61, 75)	68 (55, 75)	69 (62, 75)	0.865 ^a^
Female	76 (77.6%)	42 (85.7%)	34 (69.4%)	0.089 ^c^
Past history				
SAH	6 (6.1%)	4 (8.2%)	2 (4.1%)	0.678 ^c^
Cerebral infarction	5 (5.1%)	3 (6.1%)	2 (4.1%)	1.000 ^c^
Comorbidity				
Hypertension	46 (46.9%)	23 (46.9%)	23 (46.9%)	1.000 ^b^
Diabetes mellitus	10 (10.2%)	3 (6.1%)	7 (14.3%)	0.317 ^c^
Dyslipidemia	11 (11.2%)	8 (16.3%)	3 (6.1%)	0.199 ^c^
Current smoking	20 (20.4%)	7 (14.3%)	13 (26.5%)	0.210 ^c^
WFNS grade				
IV	55 (56.1%)	28 (57.1%)	27 (55.1%)	0.839 ^b^
V	43 (43.9%)	21 (42.9%)	22 (44.9%)	0.839 ^b^
Modified Fisher grade				
1	0 (0.0%)	0 (0.0%)	0 (0.0%)	−
2	2 (2.0%)	1 (2.0%)	1 (2.0%)	1.000 ^c^
3	27 (27.6%)	14 (28.6%)	13 (26.5%)	0.821 ^b^
4	69 (70.4%)	34 (69.4%)	35 (71.4%)	0.825 ^b^
Acute hydrocephalus	51 (52.0%)	27 (55.1%)	24 (49.0%)	0.544 ^b^
Aneurysm location				
Anterior circulation	80 (81.6%)	38 (77.6%)	42 (85.7%)	0.435 ^c^
Posterior circulation	18 (18.4%)	11 (22.4%)	7 (14.3%)	0.435 ^c^
Treatment modality				
Endovascular coiling	58 (59.2%)	29 (59.2%)	29 (59.2%)	1.000 ^b^
Surgical clipping	40 (40.8%)	20 (40.8%)	20 (40.8%)	1.000 ^b^
Coiling/clipping-related complication				
Total	30 (30.6%)	11 (22.4%)	19 (38.8%)	0.080 ^b^
Cerebral contusion	1 (1.0%)	0 (0.0%)	1 (2.0%)	1.000 ^c^
Cerebral infarction	29 (29.6%)	11 (22.4%)	18 (36.7%)	0.121 ^b^
Cerebrospinal fluid drainage				
Total	73 (74.5%)	49 (100.0%)	24 (49.0%)	<0.001 ^b^
Ventricular	47 (48.0%)	26 (53.1%)	21 (42.9%)	0.312 ^b^
Cisternal	5 (5.1%)	0 (0.0%)	5 (10.2%)	0.056 ^c^
Prophylaxis for DCI				
Cilostazol	78 (79.6%)	39 (79.6%)	39 (79.6%)	1.000 ^b^
Fasudil hydrochloride	89 (90.8%)	45 (91.8%)	44 (89.8%)	1.000 ^c^
Eicosapentaenoic acid	42 (42.9%)	19 (38.8%)	23 (46.9%)	0.414 ^b^
Statin	39 (39.8%)	25 (51.0%)	14 (28.6%)	0.023 ^b^
Low molecular dextran	49 (50.0%)	27 (55.1%)	22 (44.9%)	0.312 ^b^
Ozagrel sodium	10 (10.2%)	8 (16.3%)	2 (4.1%)	0.091 ^c^
Edaravone	24 (24.5%)	15 (30.6%)	9 (18.4%)	0.240 ^c^
Mineralocorticoid	2 (2.0%)	1 (2.0%)	1 (2.0%)	1.000 ^c^
DCI	17 (17.3%)	6 (12.2%)	11 (22.4%)	0.286 ^c^
Angiographic vasospasm	31 (31.6%)	15 (30.6%)	16 (32.7%)	0.828 ^b^
Endovascular treatment for vasospasm				
Intra-arterial fasudil hydrochloride	5 (5.1%)	3 (6.1%)	2 (4.1%)	1.000 ^c^
PTA	0 (0.0%)	0 (0.0%)	0 (0.0%)	−
Delayed cerebral infarction	26 (26.5%)	16 (32.7%)	10 (20.4%)	0.170 ^b^
On CT	12 (12.2%)	6 (12.2%)	6 (12.2%)	1.000 ^c^
On MR imaging	14 (14.3%)	10 (20.4%)	4 (8.2%)	0.147 ^c^
SDHC	46 (46.9%)	27 (55.1%)	19 (38.8%)	0.105 ^b^
90-day modified Rankin Scale 0–2	38 (38.8%)	25 (51.0%)	13 (26.5%)	0.013 ^b^

Data are numbers of patients (% of total patients per group) unless otherwise specified. *p* values: ^a^ Mann–Whitney U test, ^b^ chi-square test, or ^c^ Fisher’s exact test. CT, computed tomography; DCI, delayed cerebral ischemia; IQR, interquartile range; MR, magnetic resonance; PTA, percutaneous transluminal angioplasty; SDHC, shunt-dependent hydrocephalus; WFNS, World Federation of Neurological Surgeons.

**Table 6:** Clinical characteristics in propensity score-matched patients with poor-grade subarachnoid hemorrhage (SAH) treated with or without the triple prophylactic anti-delayed cerebral ischemia (DCI) medications consisting of cilostazol (CIL), fasudil hydrochloride (FAS), and eicosapentaenoic acid (EPA).

Variables	Overall (*n* = 204)	CIL + FAS + EPA (*n* = 102)	No CIL + FAS + EPA (*n* = 102)	*p* Value
Age (years), median (IQR)	69.0 (56.5, 77.0)	66.5 (58.0, 76.0)	70.0 (54.0, 80.0)	0.262 ^a^
Female	154 (75.5%)	75 (73.5%)	79 (77.5%)	0.515 ^b^
Past history				
SAH	8 (3.9%)	7 (6.9%)	1 (1.0%)	0.065 ^c^
Cerebral infarction	9 (4.4%)	4 (3.9%)	5 (4.9%)	1.000 ^c^
Comorbidity				
Hypertension	85 (41.7%)	43 (42.2%)	42 (41.2%)	1.000 ^b^
Diabetes mellitus	17 (8.3%)	6 (5.9%)	11 (10.8%)	0.311 ^c^
Dyslipidemia	20 (9.8%)	12 (11.8%)	8 (7.8%)	0.481 ^c^
Current smoking	40 (19.6%)	17 (16.7%)	23 (22.5%)	0.290 ^b^
WFNS grade				
IV	96 (47.1%)	52 (51.0%)	44 (43.1%)	0.262 ^b^
V	108 (52.9%)	50 (49.0%)	58 (56.9%)	0.262 ^b^
Modified Fisher grade				
1	1 (0.5%)	0 (0.0%)	1 (1.0%)	1.000 ^c^
2	3 (1.5%)	1 (1.0%)	2 (2.0%)	1.000 ^c^
3	62 (30.4%)	30 (29.4%)	32 (31.4%)	0.761 ^b^
4	138 (67.6%)	71 (69.6%)	67 (65.7%)	0.549 ^b^
Acute hydrocephalus	81 (39.7%)	43 (42.2%)	38 (37.3%)	0.474 ^b^
Aneurysm location				
Anterior circulation	182 (89.2%)	88 (86.3%)	94 (92.2%)	0.259 ^b^
Posterior circulation	22 (10.8%)	14 (13.7%)	8 (7.8%)	0.259 ^b^
Treatment modality				
Endovascular coiling	51 (25.0%)	29 (28.4%)	22 (21.6%)	0.258 ^b^
Surgical clipping	153 (75.0%)	73 (71.6%)	80 (78.4%)	0.258 ^b^
Coiling/clipping-related complication				
Total	45 (22.1%)	21 (20.6%)	24 (23.5%)	0.612 ^b^
Cerebral contusion	7 (3.4%)	4 (3.9%)	3 (2.9%)	1.000 ^c^
Cerebral infarction	38 (18.6%)	17 (16.7%)	21 (20.6%)	0.472 ^b^
Cerebrospinal fluid drainage				
Total	93 (45.6%)	46 (45.1%)	47 (46.1%)	0.888 ^b^
Ventricular	72 (35.3%)	35 (34.3%)	37 (36.3%)	0.770 ^b^
Cisternal	21 (10.3%)	12 (11.8%)	9 (8.8%)	0.646 ^c^
Spinal	28 (13.7%)	15 (14.7%)	13 (12.7%)	0.684 ^b^
Prophylaxis for DCI				
CIL alone	5 (2.5%)	0 (0.0%)	5 (4.9%)	0.059 ^c^
FAS alone	14 (6.9%)	0 (0.0%)	14 (13.7%)	<0.001 ^c^
EPA alone	4 (2.0%)	0 (0.0%)	4 (3.9%)	0.121 ^c^
CIL + FAS without EPA	54 (26.5%)	0 (0.0%)	54 (52.9%)	<0.001 ^c^
FAS + EPA without CIL	11 (5.4%)	0 (0.0%)	11 (10.8%)	<0.001 ^c^
EPA + CIL without FAS	5 (2.5%)	0 (0.0%)	5 (4.9%)	0.059 ^c^
Statin	93 (45.6%)	45 (44.1%)	48 (47.1%)	0.673 ^b^
Low molecular dextran	87 (42.6%)	46 (45.1%)	41 (40.2%)	0.571 ^b^
Ozagrel sodium	19 (9.3%)	10 (9.8%)	9 (8.8%)	1.000 ^c^
Edaravone	51 (25.0%)	26 (25.5%)	25 (24.5%)	0.872 ^b^
Mineralocorticoid	6 (2.9%)	4 (3.9%)	2 (2.0%)	0.638 ^c^
DCI	30 (14.7%)	16 (15.7%)	14 (13.7%)	0.693 ^b^
Angiographic vasospasm	59 (28.9%)	29 (28.4%)	30 (29.4%)	0.877 ^b^
Endovascular treatment for vasospasm				
Intra-arterial fasudil hydrochloride	10 (4.9%)	7 (6.9%)	3 (2.9%)	0.331 ^c^
PTA	2 (1.0%)	2 (2.0%)	0 (0.0%)	0.498 ^c^
Delayed cerebral infarction	46 (22.5%)	21 (20.6%)	25 (24.5%)	0.503 ^b^
On CT	32 (15.7%)	15 (14.7%)	17 (16.7%)	0.700 ^b^
On MR imaging	14 (6.9%)	6 (5.9%)	8 (7.8%)	0.783 ^c^
SDHC	79 (38.7%)	40 (39.2%)	39 (38.2%)	0.886 ^b^
90-day modified Rankin Scale 0–2	72 (35.3%)	43 (42.2%)	29 (28.4%)	0.040 ^b^

Data are numbers of patients (% of total patients per group) unless otherwise specified. *p* values: ^a^ Mann–Whitney U test, ^b^ chi-square test, or ^c^ Fisher’s exact test. CT, computed tomography; IQR, interquartile range; MR, magnetic resonance; PTA, percutaneous transluminal angioplasty; SDHC, shunt-dependent hydrocephalus; WFNS, World Federation of Neurological Surgeons.

## Data Availability

The data from this study will be made available to qualified investigators upon reasonable inquiry.

## Abbreviations

The following abbreviations are used in this manuscript:

aVSP	Angiographic vasospasm
CSF	Cerebrospinal fluid
CT	Computed tomography
DCI	Delayed cerebral ischemia
EPA	Eicosapentaenoic acid
IQR	Interquartile range
MR	Magnetic resonance
mRS	Modified Rankin Scale
pSEED	Prospective Registry for Searching Mediators of Neurovascular Events After Aneurysmal Subarachnoid Hemorrhage
PTA	Percutaneous transluminal angioplasty
SAH	Subarachnoid hemorrhage
SDHC	Shunt-dependent hydrocephalus
WFNS	World Federation of Neurological Surgeons

## Appendix A

pSEED Group: Department of Neurosurgery, Kuwana City Medical Center, Kuwana (Hiroshi Sakaida, Yasuyuki Umeda, Katsuhiro Tanaka, and Yoichi Miura); Mie Prefectural General Medical Center, Yokkaichi (Yusuke Kamei and Takanori Sano); Suzuka Kaisei Hospital, Suzuka (Tomohiro Araki, Masato Shiba, and Naoki Ichikawa); Suzuka Chuo General Hospital, Suzuka (Shigetoshi Shimizu, Takuro Tsuchiya, and Reona Asada); Mie University Graduate School of Medicine, Tsu (Hidenori Suzuki, Naoki Toma, Ryuta Yasuda, Fumihiro Kawakita, Yoshinari Nakatsuka, Hirofumi Nishikawa, Hideki Kanamaru, and Yotaro Kitano); Mie Chuo Medical Center, Tsu (Fujimaro Ishida, Keiji Fukazawa, and Satoru Tanioka); Matsusaka Chuo General Hospital, Matsusaka (Kazuhiko Tsuda and Yu Sato); Saiseikai Matsusaka General Hospital, Matsusaka (Hiroto Murata, and Kazuhide Hamada); Ise Red Cross Hospital, Ise (Fumitaka Miya, Hiroshi Tanemura, and Masashi Fujimoto).
